# Fatores que Impactam a Decisão de Realizar Ventriculografia Esquerda em Doença Arterial Coronariana

**DOI:** 10.36660/abc.20200217

**Published:** 2022-03-10

**Authors:** Claudia de Castro Lima Santos, Ricardo Peixoto Oliveira, Joberto Sena, Adriano Dourado Oliveira, Marcelo Gottschald Ferreira, Ademar Santos, Heitor Guissoni, José Carlos Brito, Gilson Soares Feitosa, Gilson Soares Feitosa-Filho

**Affiliations:** 1 Escola Bahiana de Medicina e Saúde Pública Salvador BA Brasil Escola Bahiana de Medicina e Saúde Pública, Salvador, BA - Brasil; 2 Hospital Santa Izabel Salvador BA Brasil Hospital Santa Izabel, Salvador, BA - Brasil

**Keywords:** Doenças Cardiovasculares, Doença Arterial Coronariana, Função Ventricular Esquerda, Ventriculografia/métodos, Angiografia Coronária/métodos, Hipertensão, Revascularização do Miocárdio/cirurgia

## Abstract

**Fundamento:**

A ventriculografia esquerda é um método invasivo para avaliar a função sistólica do ventrículo esquerdo. Depois do advento de métodos não invasivos, o seu uso tem sido questionado por resultar em algum risco para o paciente.

**Objetivos:**

Avaliar quais fatores associam-se independentemente com a decisão de realizar ventriculografia em pacientes com doença arterial coronariana.

**Métodos:**

Tratou-se de um estudo analítico, retrospectivo, avaliando prontuários eletrônicos e banco de dados e comparando 21 variáveis de interesse pré-definidas entre pacientes submetidos a cineangiocoronariografia. Foi considerado significante p < 0,05.

**Resultados:**

Avaliamos 600 pacientes consecutivos, e a ventriculografia esquerda foi realizada na maioria dos pacientes submetidos a uma cineangiocoronariografia (54%). Depois da análise multivariada, os pacientes com síndromes coronarianas crônicas ( *odds ratio* [OR] 1,72; intervalo de confiança de 95% [IC 95%]: 1,20–2,46; p < 0,01) tiveram maior chance de serem submetidos ao procedimento. Os pacientes com função ventricular conhecida (OR = 0,58; IC 95%: 0,40–0,85; p < 0,01), os revascularizados (OR 0,31; IC 95% 0,14–0,69; p < 0,01), os hipertensos (OR 0,58; IC 95%: 0,36–0,94; p = 0,02) e aqueles com maiores valores de creatinina (OR 0,42; IC 95% 0,26–0,69; p < 0,01) tiveram maior chance de não realizar ventriculografia.

**Conclusões:**

Nos pacientes submetidos a cineangiocoronariografia, o diagnóstico de síndrome coronariana crônica associou-se de modo independente com uma maior realização da técnica, enquanto ter a função ventricular previamente conhecida, ser hipertenso, ter sido submetido a revascularização cirúrgica prévia e ter valores de creatinina mais elevados associaram-se a uma maior chance de não realizar o método.

## Introdução

A ventriculografia esquerda invasiva tem sido utilizada para avaliar a função do ventrículo esquerdo por mais de 50 anos e foi, por muito tempo, o padrão-ouro.^1^ O arsenal de exames de imagem não invasivos, entretanto, vem se expandindo, oferecendo uma variedade de técnicas novas e sofisticadas. Buscando uma melhor aplicação das técnicas disponíveis, foram publicados, recentemente, critérios de uso apropriado dos métodos diagnósticos em determinadas situações clínicas. Eles estabelecem que, para avaliar a função ventricular esquerda durante a apresentação inicial de uma síndrome coronariana aguda, tanto a ecocardiografia quanto a ventriculografia são apropriadas.^2^

A realização da ventriculografia, no entanto, pode implicar em complicações. A nefropatia induzida por contraste (NIC) ocorre em, aproximadamente, 1% dos pacientes sem fatores predisponentes e em 10% a 30% daqueles com fatores de risco. Esse risco aumenta com maiores doses de contraste.^3 - 11^ Reações anafiláticas graves podem ocorrer em 0,1% dos pacientes.^12^ Outras complicações são embolização, arritmias e tamponamento, além de aumento de exposição à radiação em 30%.^12^ Atualmente, porém, a chance de complicações como reações alérgicas, sobrecarga volêmica e NIC diminuiu pelo uso de contraste não iônico, de baixa osmolaridade.^13 , 14^

Alguns estudos questionam se a avaliação invasiva pode estar sendo excessivamente indicada.^1 , 15^ Além da implicação em custo pela duplicidade de exames solicitados, há algum risco desnecessário caso outro método não invasivo esteja disponível. Esses estudos apontam, ainda, que a indicação da ventriculografia varia entre regiões geográficas e hospitais, refletindo diferença entre práticas clínicas e incerteza sobre o seu papel no diagnóstico cardiovascular.^1^ A decisão de realizar ventriculografia quando existem outros métodos não invasivos de avaliação da função ventricular disponíveis tem sido, na prática, individualizada e definida pelo cardiologista intervencionista ou pelo médico assistente.^16^

As diretrizes para síndrome coronariana preconizam a avaliação de função ventricular esquerda, recomendando um método não invasivo para essa finalidade.^17 - 26^ Apesar de considerar o exame apropriado, a literatura não estabelece, de maneira específica, o papel atual da ventriculografia e em quais situações ela deve ser priorizada em relação às técnicas não invasivas.^2^

Nosso estudo busca avaliar quais fatores estão associados à decisão de realizar ventriculografia esquerda em pacientes com doença arterial coronariana (DAC) submetidos a cineangiocoronariografia.

## Métodos

Tratou-se de um estudo analítico e retrospectivo. Foram levantados e analisados prontuários, de maneira consecutiva, até atingirmos o tamanho amostral definido de 600 pacientes submetidos a cineangiocoronariografia. Esses exames foram realizados no laboratório de hemodinâmica da Santa Casa de Misericórdia/Hospital Santa Izabel, em Salvador, no estado da Bahia, no período de 1º de janeiro de 2017 a 31 de janeiro de 2018. Consultamos, ainda, o banco de dados disponível no serviço e o sistema de laudos do setor de imagem para complementar as informações ausentes em prontuário. Como o banco de dados disponível no serviço contempla apenas pacientes submetidos a angioplastia, avaliamos apenas aqueles cuja cineangiocoronariografia resultou em posterior angioplastia.

Para o presente estudo, foram selecionados pacientes independentemente do sexo, com 18 anos ou mais, atendidos de maneira eletiva ou em caráter de urgência com suspeita de doença coronariana. Aqueles pacientes cujos registros estivessem mais de 10% incompletos em prontuário e no banco de dados seriam excluídos, porém isso não ocorreu.

Os princípios éticos que guiaram esta pesquisa estão contemplados na Resolução 466/12 do Conselho Nacional de Saúde. Este estudo foi submetido à aprovação da Comissão de Ética em Pesquisa da Santa Casa de Misericórdia/Hospital Santa Izabel, sendo aprovado com o Certificado de Apresentação para Apreciação Ética (CAAE) nº 92940318.1.0000.5520 e parecer nº 2.793.589. Por se tratar de estudo retrospectivo e baseado apenas em consulta a prontuários e banco de dados e visto que os pacientes em questão não estavam mais sendo acompanhados, foi anexado documento solicitando ao Comitê de Ética em Pesquisa dispensa do termo de consentimento livre e esclarecido, com a garantia de que os participantes não seriam identificados. Os hemodinamicistas que realizaram o exame foram identificados por uma codificação, à qual somente os pesquisadores tiveram acesso.

Esses indivíduos foram divididos em dois grupos: um composto de pacientes que foram submetidos a ventriculografia e outro de pacientes que não foram. Entre os grupos, foram coletadas e analisadas 21 variáveis candidatas à predição de indicação de ventriculografia, as quais foram selecionadas com base em estudos prévios e em nosso julgamento da plausibilidade de interferir na decisão de realizar o exame. Selecionamos variáveis relacionadas com características sociodemográficas, como sexo (feminino/masculino); idade (em anos completos); etnia (branco/não branco); índice de massa corporal (IMC); entidade financiadora do exame (se foi pública, categorizamos em Sistema Único de Saúde [SUS]; se foi particular ou convênio privado, em não SUS); história médica, como diabetes; uso de metformina; hipertensão; relato de infarto agudo do miocárdio (IAM); angioplastia coronária; e revascularização miocárdica cirúrgica e insuficiência cardíaca (ICC) prévias. Incluímos, ainda, variáveis relacionadas com a situação clínica atual e com a realização do exame, a exemplo de turno (diurno/noturno); volume de contraste (em mL); diagnóstico da admissão (síndrome coronariana crônica ou síndrome coronariana aguda); presença de instabilidade hemodinâmica; se a função ventricular esquerda era conhecida previamente por exame de imagem; qual hemodinamicista realizou a técnica; valor basal de creatinina; presença de complicações mecânicas e de DAC grave (definida neste estudo como sendo doença triarterial ou lesão de tronco de coronária esquerda).

### Cálculo do tamanho amostral

Com a finalidade de permitir a inclusão das 21 variáveis no modelo de regressão logística e considerando um mínimo de 10 pacientes por variável, estimamos um tamanho amostral mínimo de 420 pacientes. Além disso, usando por base o estudo de Hung-Hao Lee et al.,^15^ que estabeleceu entre pacientes com IAM a proporção de 44% submetidos a ventriculografia e de 56% controles, para que obtivéssemos um poder de 80% e alfa de 5%, estabelecemos um tamanho amostral de 544 pacientes. Como medida de segurança, planejamos incluir 600 pacientes.

### Análise estatística

As variáveis categóricas foram descritas como frequências e porcentagens. As contínuas, expressas em média e desvio padrão, para aquelas com distribuição simétrica, ou em medianas e intervalos interquartis (IIQ), no caso de distribuição assimétrica. A hipótese de normalidade foi verificada pelo teste de Kolmogorov-Smirnov. Na busca de variáveis preditoras de ventriculografia, realizamos uma análise univariada por intermédio dos seguintes testes: as variáveis categóricas foram comparadas por meio do teste de qui quadrado de Pearson; as contínuas com distribuição normal, utilizando o teste *t* de Student para amostras independentes; e as contínuas com distribuição anormal, com o teste não paramétrico de Mann-Whitney. As variáveis que obtiveram p < 0,05 nesses testes foram elegíveis para o modelo de regressão logística multivariada visando identificar as variáveis com associação independente depois do ajuste pelas demais. Foram considerados com significância estatística os valores de p de até 5% (p < 0,05) na análise multivariada. Para tabulação e análise dos dados, foi utilizado o programa *Statistical Package for Social Sciences* (SPSS Inc.; Chicago, IL, EUA), versão 14.0 para Windows.

## Resultados

Durante o período de 1º de janeiro de 2017 a 31 de janeiro de 2018, foram selecionados 600 pacientes submetidos a cineangiocoronariografia. Deles, 324 pacientes (54,0%) foram submetidos a ventriculografia.

Na análise das características sociodemográficas dos pacientes, 365 (60,8%) eram do sexo masculino; 479 (79,8%) se autodeclararam não brancos; 324 (54,0%) eram provenientes do SUS; a média de idade foi de 65,5±11,0 anos; e a mediana (IIQ) do IMC, de 26,0 (24,0-29,0).

Em relação às comorbidades, 248 (41,3%) dos pacientes eram diabéticos; 106 (17,7%) relataram uso de metformina; 505 (84,2%) eram portadores de hipertensão arterial; 145 (24,2%) tiveram IAM prévio; 84 (14,0%) haviam sido submetidos a angioplastia prévia; 35 (5,8%) tinham histórico de revascularização cirúrgica; e 38 (6,3%) informaram ter diagnóstico de ICC.

No tocante às variáveis relacionadas ao quadro clínico e ao procedimento, 539 (89,8%) realizaram o exame no período diurno; 202 (33,7%) pacientes tinham função ventricular conhecida; 283 (47,2%) tinham síndrome coronariana crônica; 18 (3,0%) apresentavam instabilidade hemodinâmica no momento do exame; e 54 (9%) tinham DAC grave. A mediana de uso de contraste foi 80 (60-100) mL, e a de creatinina basal, 0,8 (0,6-1,0) mg/dL. Não houve casos de complicações mecânicas nessa amostra. A realização de exames entre os hemodinamicistas variou de 12 (2,0%) a 111 (18,5%).

Observamos, na Tabela 1 , a distribuição das variáveis e sua comparação com os grupos com e sem ventriculografia. Entre as variáveis estudadas, identificamos que nove delas apresentaram significância estatística (p <0,05): convênio, idade, hipertensão arterial, revascularização cirúrgica prévia, função ventricular esquerda conhecida, diagnóstico de admissão, instabilidade hemodinâmica, creatinina basal e hemodinamicista, as quais foram incluídas no modelo de regressão logística. Na Tabela 2 , verificamos o modelo final de regressão logística, definindo os preditores independentes de indicação de ventriculografia esquerda.

**Tabela 1:** – Distribuição de variáveis e sua relação com a realização da ventriculografia

Variáveis	Total de casos N = 600	Sem ventriculografia N = 276	Com ventriculografia N= 324	Valor de p
**Sexo masculino** N (%)	365	169 (46,3)	196 (53,7)	0,86
**Etnia não branca** N (%)	479	219 (45,7)	260 (54,3)	0,83
**SUS** N (%)	324	130 (40,1)	194 (59,9)	< 0,01
Idade* (média ± DP)	65,5 ± 11,0	66,6 ± 11,5	64,7 ± 10,5	0,03
**IMC** mediana (IIQ)	26 (24-29)	25 (24-29)	26 (24-29)	0,71
**Diabetes** N (%)	248	121 (48,8)	127 (51,2)	0,28
**Uso de metformina** N (%)	106	46 (43,4)	60 (56,6)	0,59
**HAS** N (%)	505	244 (48,3)	261 (51,7)	0,01
**IAM prévio** N (%)	145	73 (50,3)	72 (49,7)	0,25
**Angioplastia prévia** N (%)	84	41 (48,8)	43 (51,2)	0,63
**Revascularização** N (%)	35	26 (74,3)	9 (25,7)	< 0,01
**ICC** N (%)	38	16 (42,1)	22 (57,9)	0,73
**Turno diurno** N (%)	539	246 (45,6)	293 (54,4)	0,68
**Função conhecida** N (%)	202	109 (54,0)	93 (46,0)	< 0,01
**Diagnóstico SCC** N (%)	283	115 (40,6)	168 (59,4)	0,01
**Instabilidade** N (%)	18	12 (66,7)	6 (33,3)	0,09
**DAC grave** N (%)	54	25 (46,3)	29 (53,7)	1,00
**Contraste** mediana (IIQ)	80 (60-100)	77 (56-100)	80 (60-100)	0,15
Creatinina** mediana (IIQ)	0,8 (0,6-1,0)	0,8 (0,7-1,1)	0,7 (0,6-0,9)	< 0,01
Hemodinamicista*** N (%)	_	_	_	< 0,01
A	27	13 (48,1)	14 (51,9)	
B	56	40 (71,4)	16 (28,6)	
C	57	12 (21,0)	45 (79,0)	
D	12	8 (66,6)	4 (33,4)	
E	21	13 (61,9)	8 (38,1)	
F	92	38 (41,3)	54 (58,7)	
G	111	46 (41,4)	65 (58,6)	
H	44	33 (75,0)	11 (25,0)	
I	30	12 (40,0)	18 (60,0)	
J	51	13 (25,5)	38 (74,5)	
L	98	47 (48,0)	51 (52,0)	

SUS: Sistema Único de Saúde, IMC: índice de massa corporal, HAS: hipertensão arterial sistêmica, ICC: insuficiência cardíaca, DAC: doença arterial coronariana, SCC: síndrome coronariana crônica, IIQ: intervalo interquartil, IAM: infarto agudo do miocárdio, DP: desvio padrão. *(N = 594), **(N = 592), ***(N = 599). Fonte: Própria autora.

**Tabela 2:** – Regressão logística ajustada pelas variáveis com p < 0,05 na análise univariada

Variáveis	OR (IC 95%)	Valor de p
Função ventricular conhecida	0,58 (0,40–0,85)	< 0,01
Diagnóstico de síndrome coronariana crônica	1,72 (1,20–2,46)	< 0,01
Revascularização cirúrgica prévia	0,31 (0,14–0,69)	< 0,01
Hipertensão arterial	0,58 (0,36–0,94)	0,02
Creatinina basal (mg/dL)	0,42 (0,26–0,69)	< 0,01

*Regressão logística ajustada por convênio, idade, hipertensão arterial, presença de revascularização cirúrgica prévia, função ventricular esquerda conhecida, diagnóstico de admissão, instabilidade hemodinâmica, creatinina basal e hemodinamicistas. OR: odds ratio; IC 95%: intervalo de confiança de 95%. Fonte: Própria autora.*

Observamos que, depois do ajuste para as demais variáveis, os pacientes cujo diagnóstico foi síndrome coronariana crônica associaram-se, de maneira independente, a uma maior chance de serem submetidos a ventriculografia, enquanto aqueles com função ventricular conhecida, com revascularização cirúrgica prévia, os hipertensos e aqueles com maiores valores de creatinina basal, a uma maior chance de não realizar o método.

## Discussão

Em nosso estudo, a ventriculografia esquerda foi realizada na maioria dos pacientes submetidos a uma cineangiocoronariografia (54%). Esse dado é compatível com aquele observado em literatura, embora haja uma variação considerável entre os serviços.^1 , 15^

Os resultados deste estudo sugerem alguns fatores que podem influenciar a decisão de realizar a ventriculografia esquerda. O paciente com diagnóstico de síndrome coronariana crônica associou-se, de maneira independente, com maior chance de realizar o método. Ter a função ventricular esquerda conhecida, ser hipertenso, ter sido submetido a revascularização cirúrgica prévia e apresentar valores aumentados de creatinina associaram-se com maior chance de não realizar a técnica. Os poucos estudos similares encontrados na literatura também observaram fatores que se correlacionaram ao uso da técnica, porém com grande variação. Parece haver, no entanto, uma tendência a se realizar o método em pacientes mais estáveis e evitá-lo naqueles com insuficiência renal.^1 , 15^

Foram menos submetidos a ventriculografia os pacientes com função ventricular conhecida e com maiores valores de creatinina. Ainda que a maioria dos pacientes tenha apresentado valores de creatinina sérica normais, encontramos uma associação significativa quando analisamos os seus valores como uma variável contínua. Essa parece ser uma decisão baseada na racionalidade, possivelmente com o objetivo de poupar o paciente de um procedimento desnecessário ou um maior volume de contraste.

Por outro lado, algumas variáveis não têm uma explicação tão intuitiva. Em nosso estudo, a ventriculografia foi mais utilizada em pacientes com síndromes coronarianas crônicas do que naqueles com síndromes coronarianas agudas, que, na teoria, poderiam precisar de uma avaliação mais imediata. Isso pode ser parcialmente explicado pelo fato de muitos pacientes estáveis realizarem o exame de maneira eletiva, trazendo consigo exames de imagem feitos fora do serviço, por vezes com dados incompletos ou qualidade questionável.

Ao avaliar o volume de contraste utilizado nos dois grupos, observamos que os pacientes submetidos a ventriculografia utilizaram apenas uma mediana de 3 mL a mais de contraste. Esse fato chamou nossa atenção, visto que, habitualmente, utiliza-se cerca de 30 mL a mais de contraste ao se realizar uma ventriculografia. Uma possível explicação seria uma tendência do cardiologista intervencionista de evitar a técnica naqueles pacientes que já haviam feito uso de um volume considerável de contraste durante a cineangiocoronariografia.

Encontramos, nas diretrizes, uma sugestão de uso da ventriculografia para ajudar a identificar a artéria culpada.^19 , 22 , 24^ Como o nosso estudo foi retrospectivo, não foi possível identificar essa situação. Outra possibilidade apontada na literatura para o uso da ventriculografia seria a avaliação de complicações mecânicas.^2^ Na nossa amostra, não houve nenhum paciente com complicação mecânica; portanto, essa situação não foi avaliada. Embora tenha havido uma variação na decisão individual de realização de ventriculografia pelos cardiologistas intervencionistas, com significância na análise univariada, não houve associação independente depois do ajuste pelas outras variáveis.

O processo de decisão médica ao realizar um exame complementar envolve vários aspectos, entre eles, o grau de evidências e informações disponíveis na literatura, as características clínicas do paciente, as diferentes circunstâncias e até as crenças do médico que indica o exame. Percebemos que as diretrizes internacionais e brasileiras não estabelecem um critério objetivo para a realização preferencial de ventriculografia esquerda invasiva em detrimento de métodos não invasivos na avaliação da função ventricular em pacientes com DAC. Acreditamos que isso se deva a uma falta de evidências na literatura, porém implica uma decisão individual do médico no momento do exame, podendo causar uma variação importante de conduta entre os centros que realizam a técnica.

Nosso estudo apresentou algumas limitações. Os dados foram coletados de maneira retrospectiva, levando a dificuldades na obtenção precisa de algumas informações. Além disso, o banco de dados contempla apenas pacientes submetidos a angioplastia. Portanto, avaliamos apenas pacientes cuja cineangiocoronariografia resultou em posterior angioplastia, o que poderia implicar algum possível viés de seleção. A alimentação dos dados foi realizada por profissionais diferentes, de diversos setores, não havendo padronização em relação ao momento da solicitação de exames laboratoriais e de imagem, dificultando a sua comparação. Para minimizar esse problema, fizemos uma busca ativa para complementar as informações por diferentes meios, como prontuário eletrônico, banco de dados e sistema de laudos do setor de imagem. No caso de pacientes estáveis admitidos para exames eletivos, a informação sobre dados demográficos, presença de comorbidades e uso de medicações prévias foi fornecida pelo próprio paciente, na sua admissão ao setor. Nos pacientes internados, a informação era registrada pelos médicos na evolução. Ainda assim, a despeito dessas limitações, nosso estudo aponta para variáveis que podem interferir na realização ou não de ventriculografia durante a execução de cateterismo, na prática clínica e fora do ambiente controlado de ensaios clínicos.

## Conclusões

Concluímos que, em pacientes com DAC submetidos a cineangiocoronariografia, o diagnóstico de síndrome coronariana crônica associou-se, de modo independente, a uma decisão mais frequente de realizar ventriculografia esquerda. Ter a função ventricular esquerda previamente conhecida por outro método, ser hipertenso, ter sido submetido a revascularização coronariana prévia e apresentar valores aumentados de creatinina associaram-se a uma maior decisão de não realizar a técnica.
