# Supplementary material for: Fatores que Impactam a Decisão de Realizar Ventriculografia Esquerda em Doença Arterial Coronariana
Source: Arq Bras Cardiol. 2022 Mar 10;118(3):607–13. [Article in Portuguese] doi: 10.36660/abc.20200217 (PMC8959033; doi:10.36660/abc.20200217)
Supplement: Translation

# Translation: Factors That Impact the Decision to Perform Left Ventriculography in Coronary Artery Disease

**Authors:** Claudia de Castro Lima Santos, Ricardo Peixoto Oliveira, Joberto Sena, Adriano Dourado Oliveira, Marcelo Gottschald Ferreira, Ademar Santos, Heitor Guissoni, José Carlos Brito, Gilson Soares Feitosa, Gilson Soares Feitosa-Filho

**Affiliations:** 1 Brazil Escola Bahiana de Medicina e Saúde Pública, Salvador, BA - Brazil; 2 Brazil Hospital Santa Izabel, Salvador, BA - Brazil

**Keywords:** Cardiovascular Diseases, Coronary Artery Disease, Ventricular Function, Left, Ventriculography/methods, Coronary Angiography/methods, Hypertension, Myocardial Revascularization/surgery

## Abstract

**Background:**

Left ventriculography is an invasive method for assessment of left ventricular systolic function. Since the advent of noninvasive methods, its use has been questioned, as it carries some risk to the patient.

**Objective:**

To assess which factors are independently associated with the decision to perform ventriculography in patients with coronary artery disease.

**Methods:**

Analytical, retrospective, database review study of electronic medical records comparing 21 predefined variables of interest among patients undergoing coronary angiography. P-values <0.05 were considered significant.

**Results:**

We evaluated 600 consecutive patients undergoing coronary angiography. Left ventriculography was performed in the majority of cases (54%). After multivariate analysis, patients with chronic coronary syndrome (OR 1.72; 95% CI: 1.20–2.46; p < 0.01) were more likely to undergo the procedure. Patients with known ventricular function (OR 0.58; 95% CI: 0.40–0.85; p < 0.01); those with a history of CABG (OR 0.31; 95% CI: 0.14–0.69; p < 0.01) or hypertension (OR 0.58; 95% CI: 0.36–0.94; p = 0.02); and those with higher creatinine levels (OR 0.42; 95% CI: 0.26–0.69; p < 0.01) had greater odds of not undergoing ventriculography.

**Conclusions:**

In patients undergoing coronary angiography, a diagnosis of chronic coronary syndrome was independently associated with greater likelihood of left ventriculography, while having previously determined ventricular function, a history of hypertension or CABG, and higher creatinine levels were associated with a decreased likelihood of undergoing this procedure.

## Introduction

Invasive left ventriculography has been used to assess left ventricular function for over 50 years, and has long been the gold standard for this purpose.^1^ However, the arsenal of noninvasive imaging modalities has been expanding, offering a variety of new and sophisticated techniques. In an attempt to improve application of available techniques, criteria for the appropriate use of diagnostic methods in certain clinical situations were recently published. These criteria state that both echocardiography and ventriculography are appropriate to assess left ventricular function during the initial presentation of an acute coronary syndrome.^2^

Ventriculography, however, can lead to complications. Contrast-induced nephropathy (CIN) occurs in approximately 1% of patients without predisposing factors, and in 10% to 30% of those with risk factors. This risk increases with higher doses of contrast.^3 - 11^ Severe anaphylactic reactions may occur in 0.1% of patients.^12^ Other complications include embolization, arrhythmias, cardiac tamponade, and a 30% increase in radiation exposure.^12^ Currently, however, the risk of complications such as allergic reactions, volume overload, and CIN has been greatly reduced by the use of nonionic, low-osmolarity contrast media.^13 , 14^

Some studies have questioned whether invasive diagnostic methods may be overused.^1 , 15^ In addition to cost concerns caused by duplication of test requests, patients are being exposed to unnecessary risk if an alternative, noninvasive method is available. Authors have also noted that the indications for ventriculography vary across geographic regions and hospitals, reflecting differences in clinical practice and uncertainty about the role of this method in cardiovascular diagnosis.^1^

In practice, the decision to perform ventriculography even when other, noninvasive methods for assessing ventricular function are available, has been made on a case-by-case basis, at the discretion of the interventional cardiologist or attending physician.^16^

Guidelines for management of coronary syndrome recommend the assessment of left ventricular function, preferably via a noninvasive method.^17 - 26^ Despite still considering ventriculography to be appropriate, the literature does not specifically establish its current role, nor in which situations it should be prioritized over noninvasive techniques.^2^

Therefore, the present study sought to assess which factors are associated with the decision to perform left ventriculography in patients with coronary artery disease undergoing coronary angiography.

## Methods

This was an analytical, retrospective study. Medical records were retrieved and analyzed consecutively until the defined sample size of patients undergoing coronary angiography (n = 600) was reached. All interventions were performed at the catheterization laboratory of Santa Casa de Misericórdia/Hospital Santa Izabel, Salvador, Bahia, from January 1, 2017 through January 31, 2018. We also consulted the department database and the imaging reporting system to obtain information missing from the electronic medical record. As the department database only includes those patients undergoing angioplasty, we evaluated only those whose coronary angiography resulted in a subsequent angioplasty.

For the present study, adult patients (18 years or older) with suspected coronary artery disease were selected regardless of gender or whether angiography was performed electively or on an emergency basis. Patients whose records were more than 10% incomplete after a review of the electronic medical record and department database would have been excluded, but no such cases occurred.

This study was conducted in compliance with the ethical principles laid out in Brazilian National Health Council Resolution 466/12. The study protocol was submitted to the Research Ethics Committee of Santa Casa de Misericórdia da Bahia/Hospital Santa Izabel for appreciation (certificate no. 92940318.1.0000.5520) and approved with opinion no. 2,793,589. Given the retrospective, chart-review design of the study and since the patients in question were no longer being actively followed, we filed for a waiver of informed consent and submitted documentation ensuring that participants would not be identified. The interventional cardiologists who performed the procedures were identified by a code, to which only the authors had access.

Participants were divided into two groups: patients who underwent ventriculography and those who did not. In these two groups, 21 candidate variables for prediction of ventriculography were collected and analyzed. These variables were selected on the basis of previous studies and of our perception of the plausibility of their interfering with the decision to perform the procedure. We selected variables related to sociodemographic characteristics, such as gender (female/male), age (in full years), ethnicity (white/nonwhite), body mass index, and payer (if public, Unified Health System – SUS; if out-of-pocket or insured, non-SUS), and medical history, such as diabetes, metformin use, hypertension, acute myocardial infarction, previous coronary angioplasty, previous coronary artery bypass grafting (CABG), or heart failure. We also included variables related to the history of present illness and the procedure itself, such as shift (day/night); contrast volume (in mL); diagnosis on admission (chronic coronary syndrome/acute coronary syndrome); presence of hemodynamic instability; whether left ventricular function was known (previously determined by imaging); interventional cardiologist who performed the procedure; baseline creatinine; presence of mechanical complications; and presence of severe coronary artery disease (defined in this study as three-vessel disease or left main coronary artery disease).

### Sample size calculation

To allow inclusion of the 21 variables of interest in the logistic regression model, and considering a minimum of 10 patients per variable, we estimated a minimum sample size of 420 patients.

Furthermore, based on a previous study by Hung-Hao Lee et al.,^15^ who reported that 44% of patients with acute myocardial infarction and 56% of controls underwent ventriculography, to obtain a statistical power of 80% at an alpha level of 5%, we established a sample size of 544 patients. For added safety, we planned to include 600 patients.

### Statistical analysis

Categorical variables were expressed as absolute and relative frequencies. Continuous variables were expressed as means and standard deviations (SD), if symmetrically distributed, or as medians and interquartile ranges (IQR) otherwise. The Kolmogorov-Smirnov test was used to confirm or reject the assumption of normality. In the search for variables predictive of ventriculography, we performed a univariate analysis. Categorical variables were compared using Pearson’s chi-square test; normally distributed continuous variables, with Student’s *t* -test for independent samples; and nonparametric variables, with the Mann–Whitney *U* test. Variables that reached p < 0.05 in these tests were carried forward into the multivariate logistic regression model, aiming to identify those variables for which an independent association remained after adjustment for the others. An alpha level of less than 5% (p < 0.05) was considered statistically significant on multivariate analysis. The Statistical Package for the Social Sciences, Version 14.0 for Windows (SPSS Inc.; Chicago, IL, USA), was used for data tabulation and analysis.

## Results

A total of 600 patients who underwent coronary angiography from January 1, 2017, to January 31, 2018, were selected. Of these, 324 patients (54.0%) underwent ventriculography.

Analysis of sociodemographic characteristics revealed that 365 patients (60.8%) were male; 479 (79.8%) self-reported their ethnicity as nonwhite; and 324 (54.0%) were covered by the Unified Health System. The mean age was 65.5 ± 11.0 years, and the median (IQR) BMI was 26 (24–29) kg/m^2^.

Regarding comorbidities, 248 patients (41.3%) had diabetes; 106 (17.7%) reported taking metformin; 505 (84.2%) had hypertension; 145 (24.2%) had a history of MI; 84 (14.0%) had undergone angioplasty in the past; 35 (5.8%) had a history of CABG; and 38 (6.3%) reported having a diagnosis of congestive heart failure (CHF).

Regarding variables related to current clinical status and the procedure, 539 patients (89.8%) underwent angiography during the day shift; 202 (33.7%) had known ventricular function; 283 (47.2%) had chronic coronary syndrome; 18 (3.0%) were hemodynamically unstable at the time of the procedure; and 54 (9%) had severe CAD. The median (IQR) contrast volume administered was 80 (60–100) mL, and the baseline creatinine was 0.8 (0.6–1.0) mg/dL. There were no cases of mechanical complications in this sample. Each of the interventional cardiologists was responsible for 12 (2.0%) to 111 (18.5%) procedures.

Table 1 shows the distribution of variables and their comparison in the groups with and without ventriculography.

**Table 1:** – Distribution of variables and their association with the decision to perform ventriculography

Variable	Total cases N=600	No ventriculography N=276	Ventriculography N=324	p-value
**Male sex** N (%)	365	169 (46.3)	196 (53.7)	0.86
**Nonwhite ethnicity** N (%)	479	219 (45.7)	260 (54.3)	0.83
**Unified Health System** N (%)	324	130 (40.1)	194 (59.9)	< 0.01
Age* mean ± SD	65.5 ± 11.0	66.6 ± 11.5	64.7 ± 10.5	0.03
**BMI Median** (IQR)	26 (24-29)	25 (24-29)	26 (24-29)	0.71
**Diabetes** N (%)	248	121 (48.8)	127 (51.2)	0.28
**On metformin** N (%)	106	46 (43.4)	60 (56.6)	0.59
**Hypertension** N (%)	505	244 (48.3)	261 (51.7)	0.01
**Prior MI** N (%)	145	73 (50.3)	72 (49.7)	0.25
**Prior angioplasty** N (%)	84	41 (48.8)	43 (51.2)	0.63
**Prior CABG** N (%)	35	26 (74.3)	9 (25.7)	< 0.01
**CHF** N (%)	38	16 (42.1)	22 (57.9)	0.73
**Day shift** N (%)	539	246 (45.6)	293 (54.4)	0.68
**Known LV function** N (%)	202	109 (54.0)	93 (46.0)	< 0.01
**Diagnosis of CCS** N (%)	283	115 (40.6)	168 (59.4)	0.01
**Instability** N (%)	18	12 (66.7)	6 (33.3)	0.09
**Severe CAD** N (%)	54	25 (46.3)	29 (53.7)	1.00
**Contrast volume** Median (IQR)	80 (60-100)	77 (56-100)	80 (60-100)	0.15
Creatinine** Median (IQR)	0.8 (0.6-1.0)	0.8 (0.7-1.1)	0.7 (0.6-0.9)	< 0.01
I nterventional cardiologist*** N (%)	_	_	_	< 0.01
A	27	13 (48.1)	14 (51.9)	
B	56	40 (71.4)	16 (28.6)	
C	57	12 (21.0)	45 (79.0)	
D	12	8 (66.6)	4 (33.4)	
E	21	13 (61.9)	8 (38.1)	
F	92	38 (41.3)	54 (58.7)	
G	111	46 (41.4)	65 (58.6)	
S	44	33 (75.0)	11 (25.0)	
I	30	12 (40.0)	18 (60.0)	
J	51	13 (25.5)	38 (74.5)	
L	98	47 (48.0)	51 (52.0)	

BMI: body mass index; CABG: coronary artery bypass grafting; CAD: coronary artery disease; CCS: chronic coronary syndrome; CHF: congestive heart failure; LV: left ventricle. *(N = 594), **(N = 592), ***(N = 599). Source: Own work.

Among the variables of interest, nine were statistically significant (p < 0.05): payer; age; hypertension; history of CABG; known left ventricular function; diagnosis on admission; hemodynamic instability; baseline creatinine; and interventional cardiologist. These variables were included in the logistic regression model. Table 2 shows the final logistic regression model, which defined the independent predictors of indication for left ventriculography.

**Table 2:** – Logistic regression adjusted for variables with p < 0.05 on univariate analysis

Variable	OR (95%CI)	p-value
Known LV function	0.58 (0.40–0.85)	<0.01
Diagnosis of chronic coronary syndrome	1.72 (1.20–2.46)	<0.01
History of CABG	0.31 (0.14–0.69)	<0.01
Hypertension	0.58 (0.36–0.94)	0.02
Baseline creatinine (mg/dL)	0.42 (0.26–0.69)	<0.01

*Logistic regression adjusted by payer, age, hypertension, history of coronary artery bypass grafting (CABG), known left ventricular (LV) function, diagnosis on admission, hemodynamic instability, baseline creatinine, and interventional cardiologist. Source: Own work.*

We observed that, after adjusting for the other variables, having a diagnosis of chronic (versus acute) coronary artery disease was independently associated with greater odds of undergoing ventriculography, while those with known ventricular function, previous CABG, hypertension, and higher baseline creatinine were more likely to not undergo ventriculography.

## Discussion

In our study, left ventriculography was performed in the majority of patients who underwent coronary angiography (54%). This is consistent with the literature, although there is substantial variation between centers.^1 , 15^

The findings of this study suggest some factors that may influence the decision to perform left ventriculography. A diagnosis of chronic coronary syndrome was independently associated with greater odds of undergoing the procedure. Having known left ventricular function, hypertension, a history of CABG, and increased baseline creatinine were associated with greater odds of not undergoing left ventriculography. The few similar studies we found in the literature also reported some factors that correlated with use of this method, albeit with great variation. However, there seems to be a trend toward performing ventriculography in more stable patients and avoiding it in those with renal failure.^1^

Patients with known ventricular function and higher creatinine levels were less likely to undergo ventriculography. Although most patients had normal serum creatinine levels, we found a significant association when we analyzed this parameter as a continuous variable. This appears to be a rationally based decision, possibly with the aim of sparing the patient from an unnecessary procedure or a higher contrast volume.

On the other hand, some variables are not so intuitively explained. In our sample, ventriculography was performed more often in patients with chronic coronary syndrome than in those with acute coronary syndromes, who, in theory, might need more immediate evaluation. This can be partially explained by the fact that many stable patients undergo the procedure electively and thus bring with them reports of past imaging performed at outside hospitals, sometimes with incomplete data or of questionable quality.

When evaluating the contrast volume used in the two groups, we found that patients undergoing ventriculography used a median of only 3 mL additional contrast. This was a striking finding, as approximately 30 mL of additional contrast is generally used when performing ventriculography as compared to angiography alone. One possible explanation would be a tendency of interventional cardiologists to avoid the technique altogether in those patients who had already received a large volume of contrast during coronary angiography.

Current guidelines suggest that ventriculography can be used to help identify the culprit artery.^19 , 22 , 24^ As our study was retrospective, we were unable to identify this particular use of the method. Another possible use of ventriculography mentioned is for evaluation of mechanical complications.^2^ In our sample, there were no patients with mechanical complications; therefore, this potential use was also not evaluated.

Despite variation in the individual decision to perform ventriculography by interventional cardiologists, which was significant on univariate analysis, there was no independent association after adjustment for other variables.

The process of deciding whether to perform a diagnostic test involves several aspects, including the degree of evidence and information available in the literature, the clinical condition of the patient, the surrounding circumstances, and even the beliefs of the physician who orders the test. Current international and Brazilian guidelines do not establish objective criteria for the preferential performance of invasive left ventriculography over noninvasive methods for the assessment of ventricular function in patients with coronary artery disease. We believe this is due to a lack of evidence in the literature; however, it forces the physician to make an individual decision at the time of angiography, which can lead to significant variation in management across centers.

This study has some limitations. Data were collected retrospectively, leading to difficulties in obtaining accurate information. In addition, the database included only patients undergoing angioplasty; therefore, we only evaluated patients whose coronary angiography resulted in a subsequent angioplasty, which may have introduced selection bias. Data were entered by different providers from different sectors, with no standardization regarding the timing of requests for laboratory tests and imaging, making comparisons difficult. To minimize this issue, we carried out an active search for supplemental information through different means, including the hospital’s electronic medical record, the catheterization laboratory database, and the radiology reporting system. In case of stable patients admitted for elective angiography, information on demographic data, presence of comorbidities, and current medications was provided by patients themselves upon admission. In hospitalized patients, this information was recorded by physicians in each patient’s progress notes.

Despite these limitations, the present study identified variables that may interfere with the decision to perform left ventriculography during cardiac catheterization in real-world clinical practice, outside the controlled environment of clinical trials.

## Conclusions

In patients with coronary artery disease undergoing coronary angiography, a diagnosis of chronic coronary syndrome was independently associated with greater odds of undergoing left ventriculography. Having known left ventricular function (determined by other imaging methods), hypertension, a history of CABG, and increased baseline creatinine were associated with greater odds of not undergoing left ventriculography.
